# Biomarkers of Osteoarthritis—A Narrative Review on Causal Links with Metabolic Syndrome

**DOI:** 10.3390/life13030730

**Published:** 2023-03-08

**Authors:** Samuel James Lynskey, Marc Julian Macaluso, Stephen D. Gill, Sean L. McGee, Richard S. Page

**Affiliations:** 1 Department of Orthopaedic Surgery, Geelong University Hospital, Geelong, VIC 3220, Australia; 2 School of Medicine, Faculty of Health, Deakin University, Waurn Ponds, Geelong, VIC 3216, Australia; 3 Barwon Health Laboratory, Barwon Health, University Hospital Geelong, Geelong, VIC 3220, Australia; 4 Barwon Centre for Orthopaedic Research and Education (BCORE), St. John of God Hospital, Deakin University, Barwon Health, Geelong, VIC 3220, Australia; 5 IMPACT—the Institute for Mental and Physical Health and Clinical Translation, School of Medicine, Deakin University, Geelong, VIC 3220, Australia

**Keywords:** metabolic syndrome, osteoarthritis, omics, transcriptomics, MetS-OA, Metabolic Syndrome associated OA

## Abstract

Development of OA (OA) is multifactorial and is strongly associated with risk factors such as aging, trauma, metabolic disorders, and obesity. Metabolic Syndrome (MetS)-associated OA, collectively coined MetS-OA, is an increasingly recognized entity in which metabolic disorders and low-grade inflammation play a key mechanistic role in the disruption of joint homeostasis and cartilage degradation. Although there have been enormous efforts to discover biomarkers of MetS and OA, studies investigating a pathophysiological link between MetS and OA are relatively limited, and no serum blood marker has proved diagnostic so far. OA biomarkers that are necessary to discriminate and diagnose early disease remain to be elicited, explained in part by limited prospective studies, and therefore limited tools available to utilize in any prognostic capacity. Biomarker validation projects have been established by the Biomarker Consortium to determine biochemical markers demonstrating predictive validity for knee OA. Given that the metabolic constituents of MetS are treatable to varying extents, it stands to reason that treating these, and monitoring such treatment, may help to mitigate deleterious links with OA development. This narrative review will describe the current state of biomarker identification and utility in OA associated with MetS. We discuss the pathophysiological mechanisms of disease according to constituent pathologies of MetS and how identification of biomarkers may guide future investigation of novel targets.

## 1. Introduction

MetS is a constellation of clinical and biochemical findings including visceral obesity, hypertension, elevated fasting glucose, and dyslipidemia [1]. Although differences exist regarding the definition, the requisite components are agreed upon by the World Health organization (WHO), the European Group for the study of Insulin Resistance (EGIR) and the International Diabetes Federation (IDF). The association of Metabolic Syndrome (MetS) with low grade systemic inflammation is increasingly recognized and designated as ‘Meta-inflammation’, a perpetual anabolic state resulting in ongoing tissue remodeling and systemic metabolic deterioration [2]. The metabolic constituents of MetS are treatable, to varying extents [3], thus it stands to reason that medical optimization and monitoring may help to mitigate deleterious links with OA development. 

A biomarker is a ‘characteristic that is objectively measured and evaluated as an indicator of normal biologic processes, pathogenic processes or pharmacologic responses to a therapeutic intervention’ [4]. Various techniques have been utilised to identify biomarkers including enzyme linked immunosorbent assays (ELISA), immunofluorescence, western blotting, immunodiffusion, polymerase chain reaction (PCR), and flow cytometry, to name a few. More recently omic technology, i.e., transcriptomics, proteomics, and metabolomics, has made efficient investigation and discovery of multiple biomarkers and biomarker profiling possible, via high throughput and unbiased analysis of a single biological sample, enabling identification of biological markers at far more minute concentrations compared to that of immunohistochemical analysis [5]. Such analyses, for example, have helped to discover an inflammatory panel of cytokines involved in knee OA [6]. Although there have been enormous efforts to discover OA specific biomarkers [7], relatively few studies have anaylsed biomarkers linked with MetS associated OA, coined MetS-OA. Delineation of the pathophysiological link between these two disease states and validation of such biomarkers remains a frontier for discovery. Current limitations in the use of biomarkers is explained in part by limited prospective studies to qualify these in any predictive capacity in order to determine disease progression. This narrative review aims to explore the discovery and utility of biomarkers linking MetS with OA and how these may guide future invesitgation of novel targets. We also aim to discuss the value of treating modifiable comorbid disease in patients with MetS-OA. 

### 1.1. Discovery of Biomarkers Pertaining to the Constituent Pathologies of MetS (HTN, Raised Fasting Glucose, Obesity, Hypercholesterolaemia) 

#### 1.1.1. Obesity and BMI 

Increased adiposity contributes to meta-inflammation by affecting local and systemic immune cell populations [8]. Accelerated OA changes have been shown in murine obesity models and meniscal or ligamentous injury; [9] further, proinflammatory endocrine mediators, rather than simply biomechanical overload, is thought to be responsible for OA development in overweight individuals [10]. Adropin is a recently discovered peptide hormone and novel biomarker of early knee OA [11]. Its activity improves insulin sensitivity, hepatic lipid metabolism and causes weight loss. Serum Adropin levels reduce proportionately to increasing Kellgren-Lawrence (KL) grade (a common radiological method of classifying OA severity [12]) [11,13]. A recent study examined the relationships between adropin level and metabolic parameters using an enzyme linked immunosorbent assay (ELISA), which showed lower serum adropin levels in Chinese T2DM patients, especially those who were overweight or obese [14]. A negative correlation has also been demonstrated between plasma adropin levels and the presence of MetS, in contrast to healthy obese and lean control subjects, which suggests that higher circulating levels may protect against MetS, even in obese patients [15]. Whether high adropin levels protect against OA remains to be seen, but utility as a marker of worsening radiographic OA is noteworthy. 

The infrapatellar fat pad in knee OA is a source of the adipokine IL-6, monocyte, chemoattractant protein-1 (MCP-1), VEGF and leptin [16], collectively promoting inflammation, migration and infiltration of monocytes and macrophages, blood vessel formation and catabolism. In murine studies, there appears to be no direct correlation between abdominal adiposity and inflammatory changes within the infrapatellar fat pad, suggesting the initiating factor for obesity induced knee OA is not likely to be mediated through infrapatellar fat pad inflammation [17]. It is more plausible that circulating adipokines are responsible for obesity induced inflammatory change in OA, as demonstrated by Richette et al., who showed that serum IL-6 levels correlated with a biomarker of cartilage turnover, Helix-II, in obese patients prior to undergoing surgically induced weight loss for knee OA [18]. Further, circulating IL-6 correlated with worsening Western Ontario and McMaster Universities Arthritis Index (WOMAC) score, a patient reported outcome measure widely validated in hip and knee OA, preoperatively. Despite worse preoperative WOMAC scores, a significant reduction in circulating IL-6 levels because of surgically induced weight loss, was not shown to correlate with symptom improvement; this is further discussed in Section 1.1.2 below [18]. 

Adiponectin is a biomarker of OA and synovitis [19,20]. To explain the pathological role adiponectin plays in OA, one study compared plasma levels between rheumatoid arthritis (RA) patients, both DMARD-naïve and chronic RA, OA, and healthy controls. Given that florid synovitis is stereotypical of RA, one might expect to find higher levels of adiponectin in the RA group. Instead, the authors found that adiponectin plasma levels were most elevated in the OA group and not explained by increased BMI, a feature of the OA cohort, which would tend to lower adiponectin levels. Systemic inflammation and circulating proinflammatory cytokines are a prominent feature of MetS, and therefore are probably not necessary for adiponectin upregulation in OA [20]. 

RBP4 is a retinol transport protein in blood, expressed in the liver and adipose tissue and closely correlated with obesity and MetS [21]. It is also produced in cartilage and synovial fluid. In cartilage cultures, RBP4 positively correlates with adipokines implicated in OA pathophysiology, including adiponectin, resistin, leptin and adipsin [21]. In animal models, RBP4 appears to be pro-inflammatory in adipose tissue, inducing cytokine secretion and systemic insulin resistance [22]. In vitro studies on human adipocytes and serum showed that elevated RBP4 levels in morbidly obese patients may promote insulin resistance through lipolysis and macrophage activation causing a pro-inflammatory cytokine release [23]. Human RPB4 also correlates with degradative enzymes including MMP-1, MMP-3, and the OA specific inflammatory marker YKL-40, suggesting RPB4 action within the synovial joint is likely to promote cartilage damage through modulation of the inflammatory process [22]. 

Other biomarkers associated with obesity include LOX-1, which through mRNA and protein expression has been shown to be upregulated in synovial fluid of patients with OA and correlated positively with BMI. However, given a lack of correlation with serum samples, practical utility as a biomarker of OA may be limited to synovial fluid sampling only [24], although subsequent investigations into its abundance in plasma may be worthwhile. LOX-1 expression is upregulated in other constituent MetS associated pathologies, including Hyperlipidaemia, Hypertension and Diabetes, limiting specificity for MetS-OA [25].

#### 1.1.2. Therapeutic Treatments Targeting Obesity-Associated OA Biomarkers

Lipopolysaccharide (LPS) is a membranous component of gram-negative bacteria commonly found in the human gut and is chronically elevated in individuals with obesity and MetS, contributing to a chronic state of low-grade inflammation [26,27]. There is some evidence that doxycycline slows the rate of joint space narrowing in osteoarthritic knees [28]. A study conducted by Huang et al. assessed the efficacy of targeting LPS in the setting of MetS-OA via treatment with a regime of doxycycline. It was observed that plasma biomarkers lipopolysaccharide binding protein (LBP) and sTLR4 were significantly associated with OA progression and severity in the knee, further supporting the role of meta-inflammation in the development and progression of knee OA [26]. Treatment with doxycycline however, did not elicit a significant effect on LBP levels, nor curtailment of OA progression [26]. 

Surgically induced weight loss has demonstrated increased serum levels of PIIANP, a biomarker of collagen type II synthesis, and a corresponding decrease in COMP, a biomarker of cartilage degradation, suggesting that weight loss may promote cartilage anabolism and decrease catabolism. Similarly, the inflammatory biomarker profile in OA improved with surgically induced weight loss, evidenced by increased serum adiponectin levels and decreased leptin, IL-6, hsCRP, orosomucoid and fibrinogen. However, the authors found no correlation between these inflammatory biomarkers and the observed clinical improvements associated with weight loss, indicating that modulation of low-grade inflammation does not explain the clinical benefits attained from surgically induced weight loss. However, the investigated biomarkers do offer potential for signifying changes in cartilage turnover [18].

A prospective cohort study evaluated the effect of a weight loss regime over 16-weeks and found that rapid substantial weight reduction resulted in decreased serum sCOMP levels, and the treatment inversely associated with urine uCTX-I and uCTX-II levels, markers of degradation of type I collagen in bone and type II collagen (although uCTX-II as a marker of cartilage turnover is debateable [29]) in cartilage, respectively; the authors argued that the latter urinary markers were associated with upregulated bone turnover accompanying weight-loss [30]. Further, the change in sCOMP after weight loss did not correlate significantly with patient symptom improvement [30]. King et al. analysed the impact of surgical and non-surgical weight loss programs in obese patients with OA, observing a relationship between weight-loss, increased adiponectin and decreased leptin levels in serum, which correlated with decreased medial tibial and medial femoral cartilage breakdown [31]. These findings highlight the effects of weight-loss on biomarkers of MetS and OA through modulation of the adipokines leptin and adiponectin, although further studies are required to validate these by correlation with patient reported outcome measure (PROM) data. Other obesity associated biomarkers worthy of prospective investigation into the effects of weight loss strategies on MetS-OA include RBP4, LOX-1 and the associated adipokines and inflammatory markers, as discussed. 

#### 1.1.3. Hyper-Cholesterolemia

Higher total and non-HDL cholesterol was associated with increased cartilage degeneration in a cross-sectional MRI study investigating MetS-OA [32]. Further, in patients with early-stage disease, namely KL I and II knee OA with and without MetS, significant increases in serum COMP, a biomarker of cartilage degeneration, in MetS-OA was noted when compared to non-MetS OA, and positively correlated with hypertriglyceridemia [33]. Serum PIIANP, a marker of cartilage regeneration [34], was not statistically different between the MetS and non-MetS OA groups, the authors maintaining that the predominant effect of MetS is catabolism, without significantly effecting repair processes [33]. These findings add further evidence to the notion that MetS tips cartilage homeostasis towards catabolism in early knee OA in a dose-response manner.

Garcia-Gil et al. in an 11-year follow-up prospective population-based study demonstrated that lower levels of hand OA were associated with higher HDL cholesterol [35]. Further, independent of a patients BMI, hyperlipidameia was documented as an independent risk factor for the development of hand OA [36] which suggests that the treatment of consitutent pathologies of MetS may play a role in curtailing disease progression. Additionally, evidence of this relationship was demonstrated in a recent meta-analysis showing that the risk of OA is higher in patients with dyslipidaemia compared to normal lipid profiles; however, the relative joint susceptibility of ‘weight bearing’ versus ‘non-weight bearing’ has not been established [37]. Distinct metabolic phenotypic subgroups of OA have been suggested as a means of classifying patients based upon the main immunometabolic anomaly driving the disease [38]. In a study investigating asymptomatic middle-aged women, it was shown that elevated or ‘high normal’ serum cholesterol and triglyceride levels were associated with the incidence of bone marrow lesions in a 2-year cross sectional study, suggesting that morphological changes in the knee can occur in the absence of clinical OA [39]. Further, raised total cholesterol and non-HDL cholesterol is also associated with worse cartilage morphology [32]. Lipid storage in articular chondrocytes is a well-known physiological phenomenon, yet this association does not reveal whether increasing accumulation of lipid with worsening histological disease severity is in an adaptive or pathologic chondrocyte response [40]. An in vitro experiment assessed human articular cartilage taken from patients undergoing TKA, demonstrating that oxidised LDL did not modify articular chondrocyte viability, and decreased the expression of pro-inflammatory and catabolic factors, namely IL-1β, IL-6, MCP-1, MMP-13, iNOS, and COX-2 gene expression, along with MMP-13 and COX-2 protein presence, under inflammatory conditions. The same study also investigated the effects of hypercholesterolaemia in a rabbit OA model and showed that cholesterol intake did not worsen cartilage degradation, suggesting that dietary cholesterol may not be responsible for deleterious effects in OA; contrariwise, mildly-oxidised LDL decreased the expression of pro-inflammatory and catabolic factors [41]. Low lipid levels promote chondrocyte commitment of skeletal progenitor cells through increased activity of the FoxO transcription factors leading to increased SOX9 production, as well as suppression of fatty acid oxidation (FAO), to allow long-term cell survival [42]. It stands to reason that increased FAO in OA in combination with high lipid availability in MetS might contribute to OA. Moreover, OA is associated with reduced SOX9 and FoxO activity [43]. Mustonen and Niieminen discuss the effects of individual fatty acids (FA) on joint tissues, acknowledging discordance in the literature between the adverse and beneficial effects of individual fatty acids in OA, suggesting that lipids may have more promise in the development of therapeutic interventions for OA than as biomarkers [44]. Saturated 16:0 and 18:0 fatty acids demonstrate mostly detrimental effects, and long-chain n-3 PUFA, especially 20:5n-3, has positive effects on joint health [44], so targeting lipid metabolism pathways may offer potential biological targets for MetS-OA [42]. However, further experimental studies are required to investigate the significance of lipid composition in OA, as well as randomised controlled trials to evaluate the efficacy of dietary fatty acid supplements in the treatment of OA [44]. 

#### 1.1.4. Therapeutic Treatments Targeting Hypercholesterolemia-Associated OA Biomarkers

Lowering systemic inflammation through treatment of MetS associated conditions with medications such as statins, SGLT-2 inhibitors, and metformin, has theoretical potential as z safe, efficacious, and non-invasive treatment option for OA, although the literature is equipoised in discerning harm and benefit. A longitudinal study concluded that the use of statins led to the slowed radiographic progression of knee OA, though statins did not lead to a significant improvement in knee OA symptomatology [45]. This is noteworthy, as radiographic progression of OA may not manifest clinically as increasing pain, the main motivation for patient presentation [46]. In contrast, a longitudinal cohort study showed that statin use for more than 5 years led to a decreased risk of developing knee pain [47]. A meta-analysis by Wang et al. concluded that there is no significant association between statin use and the incidence or progression of OA. Of note, the findings from this study were derived from observational studies, whereby causal links are difficult to establish. Further, a subsequent meta-analysis showed that statin use may be associated with increased OA development, especially at higher doses [48]. The authors further detailed in subgroup analyses an increased likelihood of hand compared with large-joint OA, suggesting that the effect of MetS and sarcopenia in the older age demographic, who are more likely to be on high dose statins, may confound interpretation of the association of high dose statins with increasing OA development [48].

To summarise the impact of dyslipidaemia in OA, increased non-HDL cholesterol affects cartilage morphology. Hypertriglyceridemia correlates with MetS-OA and increased serum COMP, a biomarker of cartilage breakdown. However, given the conjecture that exists in the literature and a possible increase in OA development at higher doses, the role of statins in MetS-OA remains highly questionable. 

#### 1.1.5. Fasting Glucose/Insulin Resistance 

The association between OA and diabetes was first described in 1961, whereby radiological changes in OA were observed in knees and feet of diabetic patients compared to matched non-diabetic controls [49]. Further, quantitative MRI assessment of knees with radiographically demonstrated KL 0–1 knee OA in patients aged 40–70 years showed that higher fasting glucose and higher HbA1c were associated with increased meniscal degeneration, a marker of degenerative change [32]. High circulating glucose is linked with meta-inflammation and may be related to the action of leptin, which normally sensitises insulin receptors in skeletal muscle, facilitating glucose utilisation [50]. The pro-inflammatory effects of leptin include stimulation of IL-6 and TNFα release from B cells [51]. In obese patients with type II diabetes, high leptin is thought to perpetuate chronic inflammation through enhanced proinflammatory Th1 cell activity, and quelled anti-inflammatory Th2 activity [52]. Leptin increases the secretion of TNF-α and IL-1, which further increase the expression of leptin in adipose tissue, creating a feedback loop that perpetuates chronic inflammation [53]. Clinically, higher leptin levels in plasma, serum and synovial fluid have been associated with increased pain, radiographic progression, bone formation biomarkers (osteocalcin and PINP), cartilage volume loss and incidence of knee OA [54], detailing this hormone as fundamental to understanding the pathophysiological link between obesity, insulin resistance and OA. 

TLR4 activity causes inflammatory effects in Type II diabetes-associated OA, and promotes catabolism in articular cartilage, as demonstrated in an in vitro study on isolates taken from patients undergoing TKR [55]. The authors suggested that TLR4 upregulation may be associated with hyperglycaemia and thus inhibition is theoretically beneficial in treating Type II diabetes mellitus-associated OA [55]. TLR4 has been reported to be involved in the synthesis and release of visfatin [21], and visfatin plasma concentrations increased in patients with obesity, type II diabetes mellitus, and MetS [56]. As a biomarker, this is noteworthy, as visfatin is catabolic to human articular chondrocytes, as demonstrated through blockade of IGF1 [57]. Franco-Trepat et al. discussed the pitfalls of using visfatin as a biomarker in OA, however, citing multiple OA-associated comorbid diseases as responsible for its systemic modulation [58]. The authors recommended that identification in synovial fluid may enhance its diagnostic appeal as an early marker of disease, as it is expressed by multiple intraarticular tissues, affording the potential to reflect the catabolic and inflammatory profile of the joint [58].

PLA2G2A is a known cartilage degradation enzyme and in serum has been shown to positively correlate with knee OA, but not to disease severity or MetS; the authors argued that this finding may be due to the inclusion of patients with end-stage disease undergoing arthroplasty [59]. Further, PLA2G2A polymorphisms have been linked to an increased risk of developing MetS and T2DM [60]; thus, it may be prudent for further investigation to corroborate a lack of correlation between PLA2G2A and MetS-OA, especially in early disease sufferers. 

Serum Metrnl is an adipokine secreted by chondrocytes and lessens lipid-induced inflammation and insulin resistance [61]. A recent study revealed that reduced serum metrnl levels were lower in the obese-OA compared with obese non-OA group; further, KL grade IV disease had significantly lower levels than lower grade disease, indicating potential applicability as a biomarker for worsening OA in obese patients. Somewhat paradoxically, however, serum metrnl levels were lower than synovial fluid levels in OA knees, suggesting independent tissue-specific regulation. In fact, serum levels are likely to reflect adipocyte secretion and synovial levels indicate chondrocyte secretion [62]. Further inference can be made that independent and local regulatory mechanisms are likely to play a role in OA progression [62]. The mechanistic details remain to be elicited. However, metrnl is expressed by the hypertrophic chondrocyte, plus a positive association between synovial fluid metrnl and insulin resistance, triglycerides level, and Total Cholesterol/HDL-Cholesterol risk ratio, as well as negatively associated with QUICKI, as shown by the insulin sensitivity index. Given this, it is thought that insulin resistance and dyslipidaemia could be involved in the hypertrophic chondrocyte phenotype [62].

Adipolin is also secreted by chondrocytes and is negatively correlated with the synovial fluid MMP-13, an ECM degradation enzyme [62]. However, given that no differences were found with KL graded severity of disease when compared with non-OA subjects, its utility as a serum biomarker of worsening disease is negligible [62].

Resistin was so named because of its role in promoting insulin resistance [63]. The link between resistin, type II diabetes mellitus and inflammation has been demonstrated, whereby higher plasm resistin and IL-6 levels were noted in patients with diabetic foot ulceration compared with patients with type II diabetes mellitus and no foot ulceration [64]. Resistin levels quantified by ELISA in synovial fluid, plasma and cartilage culture media demonstrated significant elevation in the synovial fluid of patients undergoing TKR for OA, and these levels correlated positively with inflammatory mediators IL-6, MMP-1, and MMP-3. Detracting somewhat from the meta-inflammation theory in OA progression, however, is that, surprisingly, no differences in resistin levels were seen between diabetic and non-diabetic counterparts, nor was there a correlation with BMI [65]. Further, a cross sectional study investigating blood biomarkers involved in obesity and MetS in patients undergoing TKR for end stage OA did show a correlation between FABP4, leptin and resistin with obese females; these findings were independent of MetS and not associated with knee OA severity [59]. A case control study has described Resistin as a potentially useful biomarker of worsening disease, as a positive correlation of serum resistin with WOMAC scores was demonstrated in knee OA [66]. These findings collectively demonstrate some discordance with respect to resistin’s place as a biomarker of progression in MetS-OA as examined in knee OA. Future studies should examine the correlation between resistin and MetS-OA of other joints in patients with varying stages of disease before firm conclusions are made. 

#### 1.1.6. Therapeutic Treatments Targeting Diabetes-Associated OA Biomarkers

**Figure 1:**
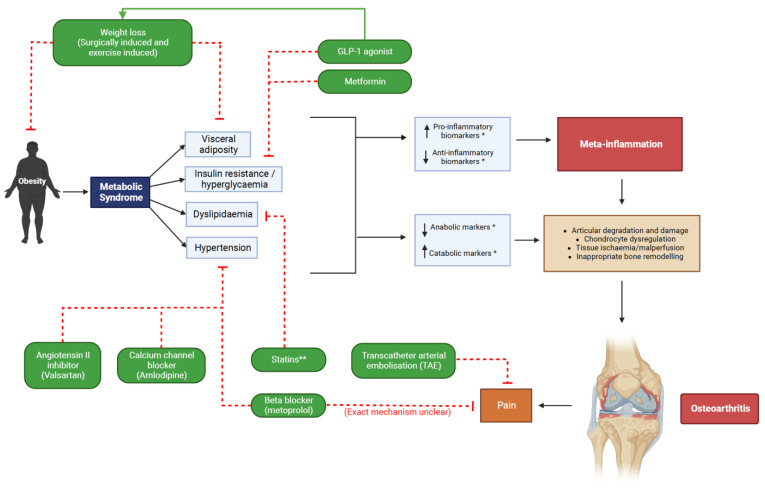
MetS-OA proposed therapeutic strategies targeting constituent pathologies. * See Table 1. for specific biomarkers. ** Higher statin dosages may be associated with progression of OA [47]. Created with BioRender.com [71].

Glucagon like peptide-1 (GLP-1) is a peptide hormone secreted by enteroendocrine cells in the intestine, alpha cells in the pancreas and in the central nervous system and, through GLP-1 agonism, regulates glucose homeostasis [67]. GLP-1 agonists have proven effective at eliciting weight-loss (See Figure 1) and are gaining popularity as a treatment for obesity, as well as for diabetes [68]. GLP-1 receptors are also found expressed in human chondrocytes and are thought to be involved in the regulation of inflammation and oxidative stress within a joint [69]. GLP-1 activation in chondrocytes has been shown to decrease inflammatory TNF-alpha, IL-6, and MMP-3/13 levels in animal studies [70]. In rat models of knee OA, the use of a GLP-1 agonist reduced joint inflammation and cartilaginous degradation [69,70], thus highlighting GLP-1 analogues as worthy of further investigation in MetS-OA.

Metformin is another MetS associated therapeutic agent that hypothetically affects MetS-OA, given its anti-inflammatory properties and subsequent effects on OA associated adipokines [72,73]. Not only is metformin effective at reducing insulin resistance and hyperglycaemia, but it also decreases circulating leptin and resistin, whilst leading to an increase in adiponectin [74]. These biomarkers are implicated in the pathogenesis of OA in MetS (see Table 1), highlighting its potential as an effective disease modifying agent in OA, as has been shown in animal models [73]. A prospective cohort study by Wang et al. concluded that the use of metformin was associated with better knee OA outcomes and prognosis through a reduced risk of requiring total knee arthroplasty (TKA) [75]. Further longitudinal studies are required to determine the mechanistic relationships between metformin and newer antidiabetic agents, and their potential role in curtailing MetS-OA progression in humans. Although many established biomarkers associated with insulin resistance act via systemic and local pro- inflammatory and catabolic mechanisms, including leptin and its association with bone formation biomarkers osteocalcin and PINP, TLR4, visfatin, PLA2G2A, metrnl, adipolin, and resistin, more investigation is required into the impact of treatment on these biomarkers in MetS-OA. 

**Table 1:** Biomarkers associated with Metabolic Syndrome as investigated in Osteoarthritis development and progression.

Biomarker	Pathophysiological Description	Association with MetS or Constituent Pathologies	Sample Type	Expression in OA/MetS (Up or Downregulation)	Reference
	Proinflammatory				
Resistin	Pro-inflammatory (increasing IL-6, MMP-1, MMP-13, MMP-3, ADAMTS-4 expression) *	Insulin resistance	SF/P/S	N.B.: Weak -ve correlation with OA severity [59] Upregulated (OA) [65,66] Upregulated (MetS-OA) [76]	[59,65,66,76]
F2RL3	Meta-inflammation *	MetS	Subject gene expression profiles obtained from GEO public repository database	Downregulated (OA) Upregulated (MetS)	[77]
GP9	Cell turnover and DNA replication *	MetS	Subject gene expression profiles obtained from GEO public repository database	Downregulated (OA) Upregulated (MetS)	[77]
ITGA2B	Platelet aggregation, inflammation *	MetS	Subject gene expression profiles obtained from GEO public repository database	Downregulated (OA) Upregulated (MetS)	[77]
Clusterin	Pain, synovitis inflammation (IL-6,8, NFκβ) and cartilage degeneration *	MetS, obesity, insulin resistance	SF/S	Upregulated (OA)	[78]
Leptin	Lipid metabolism modulation, insulin sensitivity, inflammation (IGF-1 TGF-beta1, IL-6, IL-1beta, TNF-alpha, MMP-1, MMP-3, ADAMTS 4 and 5)	MetS, insulin resistance	S/SF	Upregulated (OA) [54] Downregulated in S with weight loss (OA) [31]	[31,54]
RBP4	Upregulated by adipokines (adipsin, leptin & resistin) Meta-inflammation/synovitis (MMP1, MMP3, YKL-40) *	MetS, insulin resistance, hyperlipidaemia	P/SF/C	Upregulated (OA)	[21]
LBP	Meta-inflammation	MetS, insulin resistance	S	Upregulated (OA)	[26]
sTLR4	Immune system dysregulation—meta-inflammation Elevated uCTX-II—marker of articular cartilage degradation	MetS, meta-inflammation, insulin resistance, hyperglycaemia	S	Upregulated (OA)	[26]
LOX-1	Endothelial damage, inflammation, articular cartilage catabolism (MMP-3)	Hypercholesterolaemia, atherosclerosis, hypertension, insulin resistance, T2DM	C, SF	Upregulated (OA)	[24]
Chemerin	Endothelial & synovial inflammation (cytokine release from synovial fibroblasts) Articular cartilage catabolism	MetS, atherosclerosis, obesity	SF/ST	Upregulated (OA)	[79]
SERPINE2	Meta-inflammation *	Obesity	ST	Upregulated (OA)	[80]
ITIH5	Cell differentiation, ECM stabilisation *	Obesity	ST	Downregulated (OA) Upregulated (MetS)	[80]
IL-6	Endothelial damage, meta-inflammation—adipose and articular fat pads Cartilage degradation (Helix II—marker for degradation)	MetS, insulin resistance, obesity, meta-inflammation	S	Upregulated—associated with worse WOMAC scores in knee OA Downregulated with surgical weight loss—no correlation with clinical outcomes	[18]
Visfatin	Chronic inflammation (IL-1, IL-6, TNF-α)—secreted by white adipose	Insulin resistance, Type II diabetes, obesity	SF	Upregulated (OA)	[58]
oxLDL	Binds chondrocyte LOX—oxidative stress, cartilage degeneration and inflammation	Hypercholesterolaemia, atherosclerosis	C	Upregulated (OA)	[24]
	Anti-inflammatory				
ELOVL7	Fatty acid elongation and accumulation of lipids *	MetS, hypercholesterolaemia and dyslipidaemia	Subject gene expression profiles obtained from Gene Expression Omnibus (GEO) public repository database	Downregulated (OA) Upregulated (MetS)	[77]
Metrnl	Inflammation and cartilage catabolism ^#^ (decreased PPAR-γ) *	Insulin resistance ^#^ Hyperlipidaemia ^#^	S/SF	Downregulated (In serum) Upregulated (In SF)	[62]
HDL-C	Dysregulated lipid metabolism *	Obesity ^#^, MetS ^#^ and dyslipidaemia ^#^	SF	Downregulated (OA)	[81]
ApoA1	Dysregulated lipid metabolism *	Obesity ^#^, MetS ^#^ and dyslipidaemia ^#^	SF	Downregulated (OA)	[81]
Adiponectin	Anti-inflammatory: - TNF-α inhibition - Inhibits macrophage phenotype differentiation M2 > M1 (pro-inflammatory) Anti-catabolic (inhibits MMP-2, MMP-13)	Central obesity ^#^, MetS ^#^, insulin sensitivity	P (Laurberg)	Upregulated (OA)	[19,20]
Adropin	Inhibition of pro-inflammatory cytokines (TNF-α) *	Hypertension ^#^, insulin resistance ^#^, type II diabetes ^#^	S	Downregulated (OA)	[11]
	Anti-inflammatory/Anti-catabolic				
Adipolin	Anti-catabolic (MMP-13 ^#^) *	Insulin sensitivity	S/SF	Downregulated (OA) ns	[62]
	Catabolic				
COMP	Biomarker of cartilage loss Prognostic indicator of joint damage, pain & stiffness WOMAC score	MetS, Obesity and hyperinsulinaemia	S	Downregulated (OA) with surgical weight loss	[18]
	Anabolic				
PIIANP	Biomarker of cartilage anabolism *	No correlation with MetS	S	Upregulated (OA) with surgical weight loss	[18]

S: Serum, SF: Synovial fluid, ST: Synovial tissue, U: Urine, C: Cartilage tissue, Ch: Chondrocytes, ns: not statistically significant, * Exact mechanism in MetS-OA is unknown, ^#^ inverse association.

#### 1.1.7. Hypertension

Evidence for a causal link between hypertension and OA is limited and the assumption of such an association is derived primarily from epidemiological data [82]. A recent meta-analysis depicted a BMI-independent association between radiographic knee OA and hypertension, demonstrating hypertension as detrimental to OA progression [83]. Arterial calcification itself has been shown to be a biomarker of spine OA, with abdominal aortic calcification shown to correlate with anterior lumbar osteophyte formation, but not hand osteophyte formation [84]. It has been theorised that hypertension may induce subchondral ischemia, thereby altering the activity of VEGF. Increased plasma [85] and synovial fluid [86] levels of VEGF correlated with OA severity and progression in human studies respectively. VEGF is proinflammatory and acts via autocrine stimulation, enhancing expression of MMP-1, MMP-3, and MMP-13, whereas it reduces TIMP-2, a natural inhibitor of MMPs [87]. A recent meta-analysis demonstrated a 62% increased odds of radiographic knee OA in hypertensive patients adjusting for BMI, and the relationship was stronger for women (127% increased odds) than men (45% increased odds) [83]. Of note, the increased odds of developing hand OA was only 19% suggesting that the effects of altered vascularity to subchondral bone in combination with other pathologic biomechanics may be responsible for radiographic changes, more so than either risk factor in isolation [83]. Indeed, long-distance runners, when compared to healthy aged match non-running controls, did not suffer an increased risk of radiographic knee OA progression [88]. 

Intraosseous hypertension has been demonstrated in the femur of patients with rest pain and OA [89]. Marrow hypertension, oedema and micronecrosis alter the blood flow to subchondral bone which is likely to have deleterious effects on cartilage by diminishing nutrient supply to avascular regions [90]. In contrast to this, transcatheter arterial embolization (TAE) (See Figure 1) has recently emerged as potentially efficacious in treating pain from OA by ablating periarticular neovessels [91]. The goal of this technique is to return the microvascular environment to baseline state, disrupting inflammatory processes and aiming to limit disease progression [92]. It may be that neovascularity increases inflammation and pain in OA, but well-designed longitudinal studies are required to determine the relative contribution of hypertension to this phenomenon, and the benefit or harm associated with ablation of this neovascular network to the osteoarthritic process. 

Indirect biomarkers of MetS include Ghrelin, which is vascular protective through antagonism of vasoconstrictors and stimulation of lipolysis. Ghrelin is a peptide secreted X/A-like gastric cell inducing an orexigenic state and suppressing inflammation [93]. In keeping with the correlation of lower adropin levels in type II diabetes mellitus, worsening knee OA and MetS, lower levels have also been demonstrated in patients with hypertension [94]. 

#### 1.1.8. Therapeutic Treatments Targeting Hypertension-Associated OA Biomarkers

Serum adropin levels were demonstrated to increase after 12 weeks of treatment with valsartan or amlodipine [95]. These findings illustrated a causal link between serum adropin and antihypertensive medication; however, correlation with PROM data in MetS-OA is needed to determine the relationship between serum adropin levels and OA symptomatology in response to antihypertensive treatment modalities.

An observational study conducted by Valdes et al., 2017 observed that beta-blockers such as metoprolol were associated with a statistically significant decrease in OA associated joint pain and associated analgesia use [96]. Furthermore, implementation of a calcium channel blocker showed some efficacy in minimising the development of OA in animal models [97]. In addition to potential chondroprotective effects, some antihypertensive medications have been demonstrated to reduce inflammation [98]. The importance of anti-inflammation to chondroprotection and subchondral bone function was demonstrated after a single intra-articular injection of the TNF-α inhibitor Adalimumab, which revealed improved pain visual analogue, and improved WOMAC scores in patients with severe knee OA. [99] Systemic HTN impairs subchondral bone perfusion, disrupting the angiogenic–osteogenic coupling and thus the integrity of the bone-cartilage functional unit. [100] Further, the renin–angiotensin, endothelin and Wnt–β-catenin signaling pathways alters articular chondrocyte phenotype leading to catabolism [100]. Optimising blood flow to subchondral bone by treating HTN reduces both inflammation and catabolism (See Figure 1); however, future studies should determine the degree to which normalisation of blood pressure effects MetS-OA by correlating with pain, functional scores and biomarkers of cartilage breakdown.

### 1.2. Discussion 

MetS is considered a singular disorder; however, to explore the current state of biomarker discovery in MetS-OA, the literature amassed in this review addresses known biomarkers of MetS as well as its constituent pathologies, such as visceral obesity, hypertension, elevated fasting glucose, and dyslipidemia, and how these relate to biomarker expression in OA. Pragmatically this enabled a detailed discussion not only of the association between biomarkers of MetS and constituent pathologies, but also of the plausible mechanistic relationships driving this observation. On the latter, much work must be done. There is a simultaneous need to undertake targeted validation and exploratory works into MetS-OA. Mechanistic molecular studies are required to further elucidate the hypothesized interplay between constituent pathologies of MetS and OA discussed here. Relatively little is known about the role of HTN in OA, and the literature is equipoised to discern the role of individual fatty acids in OA pathophysiology. Further enquiry into dyslipidaemia and meta-inflammation will likely demonstrate higher yield in the discovery of novel treatments than prognostic applicability, as clinically relevant biomarkers. Future pathophysiological studies exploring mechanisms of disease should guide investigation of novel treatments and drug-repurposing in MetS-OA. Alternatively, as demonstrated by Huang et al., by utilising already established datasets from well designed multicentre, randomized, double-blind, placebo-controlled trials with large sample sizes, targeted biomarker validation studies can be performed, as was shown with LBP, identified as a mediator of doxy-cyline treatment effects on the structural progression of OA [26,101] Collaborative approaches are required to distil clinically relevant research questions and to enhance the efficiency of scientific enquiry. 

OA pathophysiology involves the whole synovial joint complex including articular cartilage, synovium and subchondral bone, with complex disharmony between destruction and maladaptive repair [102] and, as observed by Sobieh et al., tissue and context-dependant regulatory mechanisms for biomarker production. Specifically, the synovial fluid metrnl is produced by chondrocytes, as compared to serum metrnl, which is likely to come from adipocytes [62]. Consequently, collaborative research needs to determine the most applicable and appropriate tissue type prior to omic exploratory enquiry, in order to best answer the clinical question at hand. Further, given that the genetic loci associated with hip and knee OA are different [103], to determine the joint susceptibility and biomarker expression profile, future studies should specifically address how MetS affects biomarker expression in both weight bearing and non-weight bearing joints. 

Some reports have demonstrated utility at correlating the expression of inflammatory biomarkers with OA severity, namely adropin (downregulated), LPB, sTLR4 and Resistin (weakly) [11,13,26,66]. Further, Ungsudechachai et al. suggested that clusterin possibly mediates obesity-associated synovitis, and plasma levels could potentially be used to differentiate KL stage 3 and 4 from KL stage 2 with a calculated sensitivity of 71.4% and a specificity of 73.3% [78]. Bartels et al. acknowledge large variations in KL grading of OA, with higher grade disease potentially failing to demonstrate changes in tissue turnover, due to the nature of cell content in severe disease [30]. As such, interventions targeting treatments for MetS-OA should stratify and group disease to evaluate markers of tissue turnover and clinical response to interventions, especially to determine the potential impact on the natural history of disease for patient with MetS and mild OA. 

Many studies have discussed utilization of biological samples from patients undergoing arthroplasty, whereby subtle and early changes in catabolic and inflammatory pathways may be obscured by the ‘noise’ associated with disharmonious activity of pro- and anti- inflammatory and catabolic pathways observed in advanced OA. Given this, inflammatory biomarkers involved in MetS, tend to be upregulated in OA with few exceptions, including GP9 and ITGA2B, both of which demonstrated downregulation in OA but upregulation in MetS. It was suggested that a novel mechanism might explain the role of MetS in OA progression, connecting OA cartilage regeneration with impaired cell proliferation and concomitant cell-cycle arrest (see Table 1) [77]. This further illustrates the need for future validation studies to consider patients with MetS and preclinical or mild disease. 

### 1.3. Conclusions

This review highlights multiple biomarkers linking MetS with OA, adding to the weight of evidence of MetS-OA as an entity deserving of ongoing exploration. The pervading question underpinning Mets-OA as an important research frontier is whether meta-inflammation is reversible. To capitalise on the impactful work detailed in this review will require simultaneous mechanistic, exploratory, and clinically based enquiry. There is a need to better determine the pathophysiological mechanisms of meta-inflammation in MetS-OA progression. Further, it is imperative to determine to what degree optimal treatment for the four central features of MetS (visceral obesity, hypertension, elevated fasting glucose, and dyslipidaemia) improves clinical and radiological outcomes in MetS-OA, and the utility of biomarkers in aiding diagnosis of preclinical disease, prognostication and treatment for established and novel modalities. 

## Data Availability

Not applicable.

## Abbreviations

Abbreviation	Definition
ApoA1	Apolipoprotein A1
COMP	Cartilage oligomeric matrix protein
COX-2	Cyclooxygenase-2
ECM ELOVL7	Extracellular matrix Elongation of very long chain fatty acids protein 7
F2RL3	F2R like thrombin or trypsin receptor 3
FABP4	Fatty acid binding protein 4
FoxO	Forkhead box O
GP9	Gene encoding for platelet glycoprotein IX
HDL-C	High density lipoprotein cholesterol
IL-1β	Interleukin-1-beta
IL-6	Interleukin 6
iNOS	Inducible nitric oxide synthase
ITIH5	Inter-alpha-trypsin inhibitor heavy chain family, member 5
LOX-1	Lectin-like oxidised low-density lipoprotein receptor 1
LPS	Lipopolysaccharide
MCP-1	Monocyte, chemoattractant protein 1
Metrnl	Meteorin-like protein
MMP-1	Matrix metalloproteinase 1
MMP-13	Matrix metalloproteinase 13
MMP-3	Matrix metalloproteinase 3
NGF oxLDL	Nerve growth factor Oxidised Low-density-lipoprotein
PIIANP	Serum N-terminal pro-peptide of type IIA collagen
PLA2G2A	Phospholipase A2, membrane associated
PUFA	Polyunsaturated fatty acid
QUICKI	Quantitative insulin-sensitivity check index
RBP4	Retinol binding protein 4
SGLT-2	Sodium-glucose cotransporter 2
SOX9	SRY-Box transcription factor 9
sTLR4	Serum Toll-like receptor 4
TAE	Transcatheter arterial embolization
TIMP-2	Tissue inhibitor of metalloproteinase 2
TLR4	Toll-like receptor 4
TNFα	Tumour necrosis factor alpha
uCTX-I	Urinary Crosslinked C-Telopeptide of Type I Collagen
uCTX-II	Urinary Crosslinked C-Telopeptide of Type II Collagen
YKL-40	Chitinase-3-like protein 1 (derived from three N-terminal amino acids present on the secreted form and its molecular mass)
